# Effects of Transcutaneous Spinal Direct Current Stimulation (tsDCS) in Patients With Chronic Pain: A Clinical and Neurophysiological Study

**DOI:** 10.3389/fneur.2021.695910

**Published:** 2021-09-06

**Authors:** Matteo Guidetti, Roberta Ferrucci, Maurizio Vergari, Giada Aglieco, Anisa Naci, Sara Versace, Kevin Pacheco-Barrios, Stefano Giannoni-Luza, Sergio Barbieri, Alberto Priori, Tommaso Bocci

**Affiliations:** ^1^“Aldo Ravelli” Center for Neurotechnology and Experimental Brain Therapeutics, Department of Health Sciences, University of Milan, Milan, Italy; ^2^Department of Electronics, Information and Bioengineering, Politecnico di Milano, Milan, Italy; ^3^Azienda Socio-Sanitaria Territoriale-Santi Paolo e Carlo University Hospital, Milan, Italy; ^4^Neurophysiology Unit, Foundation Istituto di Ricerca e Cura a Carattere Scientifico Ca' Granda Ospedale Maggiore Policlinico, Milan, Italy; ^5^Neuromodulation Center, Spaulding Rehabilitation Hospital, Boston, MA, United States; ^6^Center for Clinical Research Learning, Massachusetts General Hospital, Boston, MA, United States; ^7^Unidad de Investigación para la Generación y Síntesis de Evidencias en Salud, Universidad San Ignacio de Loyola, Lima, Peru

**Keywords:** chronic pain, treatment, transcutaneous spinal direct current stimulation, tsDCS, non-invasive brain stimulation, neurophysiology

## Abstract

**Background and Aims:** Chronic pain is a complex clinical condition, often devastating for patients and unmanageable with pharmacological treatments. Converging evidence suggests that transcutaneous spinal Direct Current Stimulation (tsDCS) might represent a complementary therapy in managing chronic pain. In this randomized, double-blind and sham-controlled crossover study, we assessed tsDCS effects in chronic pain patients.

**Methods:** Sixteen patients (aged 65.06 ± 16.16 years, eight women) with chronic pain of different etiology underwent sham and anodal tsDCS (anode over the tenth thoracic vertebra, cathode over the somatosensory cortical area: 2.5 mA, 20 min, 5 days for 1 week). As outcomes, we considered the Visual Analog Scale (VAS), the Neuropathic Pain Symptom Inventory (NPSI), and the components of the lower limb flexion reflex (LLFR), i.e., RIII threshold, RII latency and area, RIII latency and area, and flexion reflex (FR) total area. Assessments were conducted before (T0), immediately at the end of the treatment (T1), after 1 week (T2) and 1 month (T3).

**Results:** Compared to sham, anodal tsDCS reduced RIII area at T2 (*p* = 0.0043) and T3 (*p* = 0.0012); similarly, FR total area was reduced at T3 (*p* = 0.03). Clinically, anodal tsDCS dampened VAS at T3 (*p* = 0.015), and NPSI scores at T1 (*p* = 0.0012), and T3 (*p* = 0.0015), whereas sham condition left them unchanged. Changes in VAS and NPSI scores linearly correlated with the reduction in LLFR areas (*p* = 0.0004).

**Conclusions:** Our findings suggest that tsDCS could modulate nociceptive processing and pain perception in chronic pain syndromes.

## Introduction

Chronic pain is one of the most intractable clinical problems faced by clinicians and can be devastating for patients (1). Central pain cannot be fully explained based on somatic or neuropathic processes, relying on changes both in ascending and descending nociceptive pathways (2). Different factors seem to contribute to the chronization of pain, both at cortical and spinal level, including functional reorganization of the cortical sensorimotor maps, thalamo-cortical dysrhythmia and spinal “phenotypic switch” in the expression of neuropeptides (3–6). The complexity and heterogeneity of these maladaptive mechanisms make pharmacological treatments often inappropriate.

Several non-invasive brain stimulation (NIBS) techniques have given evidence to improve pain; cerebellar transcranial direct current stimulation (tDCS), a recent technique of stimulation, has shown promising results for pain reduction (7, 8), while high-frequency repetitive Transcranial Magnetic Stimulation (rTMS) and anodal tDCS applied over the contralateral primary motor cortex (M1) have been suggested as effective non-pharmacological tools for chronic pain (9–13). Among NIBS techniques, a growing body of literature indicate that transcutaneous spinal direct current stimulation (tsDCS) modulates both spinal and supra-spinal excitability (14, 15). Indeed, in the last decades, different studies applied DC stimulation over the thoracic and cervical spinal cord to modulate spinal pathways (16–18) in humans, providing compelling evidence that tsDCS affects somatosensory, motor, and nociceptive spinal circuits (14, 15, 19–21). tsDCS changes not only the conductive but also trans-synaptic efficacy of spinal neurons (21, 22). Although the mechanisms of action are still to be elucidated, tsDCS likely modulates also the supra-spinal excitability, probably in a polarity-dependent manner (23, 24), with anodal tsDCS leading to an impaired intracortical excitability and a functional disconnection between hemispheres (24, 25). In this scenario, Cogiamanian et al. (19) found that anodal tsDCS induced lower limb flexion reflex (LLFR) depression in healthy subjects and confirmed that the non-invasive spinal neuromodulatory technique could modulate central nociceptive signal transmission; therefore, it might represent a complementary therapy to drugs and invasive spinal cord stimulation (SCS) in managing chronic pain.

LLFR is a polysynaptic and multi-segmental spinal reflex that induces a complex flexion synergy of the stimulated limb; it has two main components, formally named RII and RIII, which derive from the activation of the cutaneous A-beta and nociceptive A-delta fibers, respectively (26–28). Guidelines recommend the RIII reflex as the most reliable nociceptive reflex for the assessment of pain treatment efficacy (29); it has also been proposed as a neural window onto the spinal mechanisms activated during locomotion, both in animals (30–32) and in humans (28).

To the best of our knowledge, no study has evaluated to date whether tsDCS modulates the RIII reflex, as a measure of the central nociceptive processing in chronic pain patients. Our aim was to evaluate the use of tsDCS, applied over the spinal cord (spinous process of the tenth thoracic vertebra, the anode) and primary somatosensory cortex (S1, the cathode), as non-pharmacological treatment in patients with chronic pain of different etiologies, by monitoring the effects on the LLFR and clinical scales, as assessed by the Visual Analog Scale (VAS) and the Neuropathic Pain Symptom Inventory (NPSI).

## Materials and Methods

### Subjects

Sixteen patients (aged 65.06 ± 16.16 years, 8 women) with chronic pain have been enrolled in our study. Demographic features are summarized in Table 1. Inclusion criteria were: (1) age 18–70 years; (2) normal score (>24) at the Mini-Mental State Examination; (3) stable presence of pain for at least 5 years, (4) no coexistence of major neurologic, neuropsychological, and psychiatric diseases as confirmed by clinical history and anamnestic interview; and (5) stable pharmacological therapy during the month before the inclusion. Exclusion criteria were as follows: (1) participants who started new medical treatments or physiotherapy within 1 month prior of the recruitment; (2) participants had any history/current signs or symptoms of neurologic or psychiatric disorders; (3) deep brain stimulation implanted patients; (4) pregnancy; and (5) carriers of spinal cord stimulators. The study protocol followed to the Declaration of Helsinki and was approved by the Ethics Committee of the IRCCS Ca' Granda Foundation—Maggiore Policlinico Hospital of Milan. All subjects gave written informed consents before the participation.

**Table 1:** Demographic characteristics of the subjects.

**Patient no**.	**Etiology of pain**	**Timeline of intervention**	**Pharmacological treatment**	**Baseline VAS**	**Baseline NPSI**
1	Post-herpes	Anodal/Sham	Pregabalin	71/76	41/51
2	Lumbosacral Radiculopathy	Sham/Anodal	Oxycodone/naloxone	63/45	13/19
3	Lumbosacral Radiculopathy	Anodal/Sham	None	57/52	67/40
4	Idiopathic	Anodal/Sham	Methylprednisolone	89/90	17/22
5	Diabetes	Sham/Anodal	None	23/61	12/36
6	Lumbosacral Radiculopathy	Anodal/Sham	Etoricoxib	98/61	61/36
7	Lumbosacral Radiculopathy	Sham/Anodal	Clonazepam	34/61	43/36
8	Lumbosacral Radiculopathy	Anodal/Sham	None	75/21	40/12
9	Lumbosacral Radiculopathy	Anodal/Sham	None	44/28	48/42
10	Lumbosacral Radiculopathy	Anodal/Sham	None	100/64	47/40
11	Multiple Sclerosis	Sham/Anodal	Etoricoxib and Baclofen	100/69	81/70
12	Lumbosacral Radiculopathy	Anodal/Sham	None	70/85	56/34
13	Diabetes	Sham/Anodal	None	40/61	44/36
14	PLMT	Sham/Anodal	Levetiracetam	95/97	58/57
15	Lumbosacral Radiculopathy	Sham/Anodal	Oxycodone/naloxone	36/40	20/13
16	Post-herpes	Sham/Anodal	Pregabalin	66/61	43/36

*PLMT, painful legs and moving toes syndrome; VAS, Visual Analog Scale; NPSI, Neuropathic Pain Symptom Inventory*.

### Study Design and Experimental Procedures

**Figure 1:**
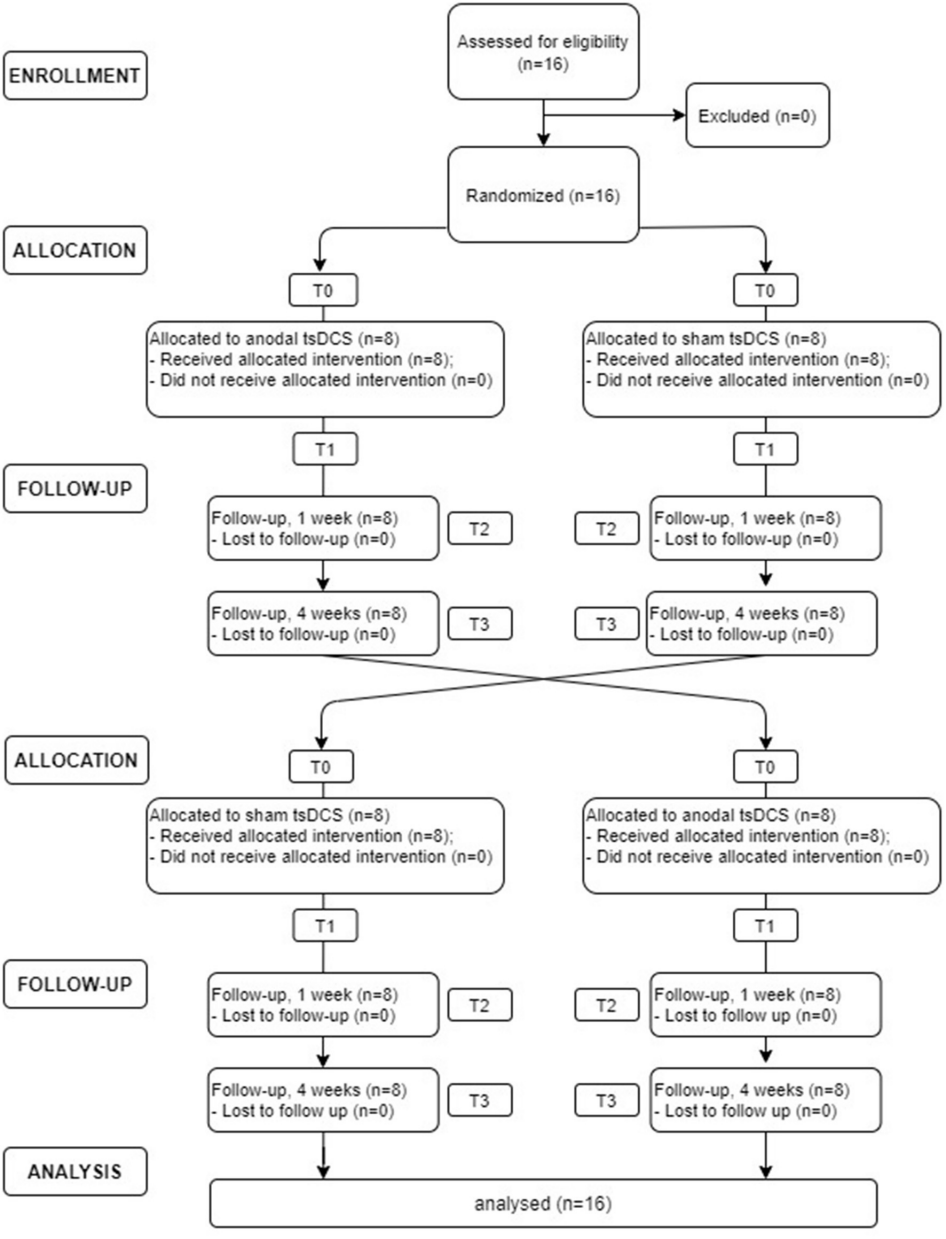
Flowchart diagram depicting the flow of participants through study.

In this randomized, double-blind, sham-controlled crossover study, each patient underwent sham and anodal tsDCS (2.5 mA for 20 min, once a day for 5 days). Participants were assigned randomly to either group A (active tsDCS first) or group B (sham tsDCS first), using a random number generator. At the next treatment, patients were crossed over to the opposite treatment. Due to the potential carryover effect from crossover design, a washout period of at least 4 weeks was provided between treatments. Clinical (VAS and NPSI) and neurophysiological (LLFR) outcome were assessed before (T0) and at the end (T1) of the 5-day treatment and 1 week (T2) and 1 month (T3) after the completion of the stimulation protocol (see Figure 1). Both subjects and assessors were blinded to the stimulation condition.

### Transcutaneous Spinal Direct Current Stimulation

The application of spinal DCS followed key technical features already verified in literature (14, 19, 33, 34). Briefly, patients were asked to lie down on a couch, and tsDCS (2.5 mA, 20 min) was delivered by a programmable stimulator (HDCStim™, Newronika, Italy) connected to a pair of rectangular electrodes; the anode was placed over the spinous process of the tenth thoracic vertebra (from the 10th to 12th vertebra, with the major axis parallel to spinal cord), and the cathode over the somatosensory cortical area (2 cm rear of CZ, according to 10/20 EEG system). The tsDCS electrodes were rectangular pieces of saline-soaked synthetic sponge (7 × 8 cm, 56 cm^2^). We applied a current density of 0.035 mA/cm^2^ and delivered a total charge density of 42.8 mC/cm^2^, below the threshold values reported for tissue damage (35, 36). At the onset of tsDCS, the current was increased for 30 s, and at the offset, it was decreased for 30 s in a ramp-like manner, a method shown to achieve a good level of blinding among sessions (37, 38). For a sham tsDCS, the current was turned on for 5 s and then turned off in a ramp-shaped fashion, thus inducing skin sensations indistinguishable from anodal tsDCS. Assessors and patients were blinded to the tsDCS protocol; patients did not discriminate between anodal and sham condition, neither reported adverse effects as evidenced by the questionnaire developed by Brunoni et al., which was administered to each patient (12).

### LLFR Recording

Lower limb flexion reflex (LLFR) was recorded from the left lower limb with patients in a prone position on a bed in a prepared room, with the ankle flexed at 90°. Ag/AgCl surface-capping electrodes placed with patches were used to guarantee the signal was recorded at the same position during all the experiments and ensure a reliable registration. Furthermore, an electro-conductive gel was used under the stimulating electrodes to optimize the passage of the currents and increase patient compliance. The RIII reflex was evoked and recorded from the left lower limb, as described in detail elsewhere (39, 40). Briefly, the recording electrode was placed on the short head of the biceps femoris; the reference electrode was placed on the capital of the fibula; and the earth electrode was placed on gastrocnemius muscle, in an intermediate position between the stimulation and registration site. The stimulation was percutaneous and ipsilateral to the recording side; the electrodes were placed at the retro-malleolar pathway of the sural nerve. Five responses were recorded after a stimulus of 1 ms duration, elapsed by 5–15 interstimulus intervals (ISI) to avoid habituation (27, 41). The signal was subsequently corrected and averaged, and the following parameters were quantified: the RIII threshold (mA), i.e., the minimum current intensity at which the reflection appears, the latencies (ms), and the area (mV^*^ms) of the main components of the FR (RII and RIII) were assessed. Stimuli with increased intensities were delivered with an interval of 2.5 mA at a time to obtain a repeatable response in three consecutive recordings, with a minimum width of at least 50 μV. The RII response, originated from the sensory activation of the nerve fibers, usually appeared with a lower stimulus intensity compared to RIII, with latency between 40 and 80 ms. Once the RII response was obtained, the intensity of the stimulus was increased until the RIII response appeared. Five responses of FR were recorded with a stimulus intensity set at the threshold 20% higher than RIII. Since the FR is variable, the recording was kept in a constant time of day for all the evaluation time points (T0, T1, T2, and T3). The voluntary EMG activity at rest was monitored by video-audio feedback to ensure the lack of significant muscle contraction during experimental sessions.

### Clinical Evaluation

The Visual Analog Scale (VAS) (42) and Neuropathic Pain Symptom Inventory (NPSI) (43) were performed at T0, T1, T2, and T3. The VAS is represented by a 10-cm-long line in which one end indicates the absence of pain (0), and the other end corresponds to the worst pain perceived (10). Each patient was asked to indicate the intensity of his/her level of pain with a line (variable between 0 and 10) at that precise moment. NPSI consists of 12 questions to investigate the different types of pain that a patient feels with a score between 0 and 10 (except for two questions in which the maximum assignable score is 5). The final score was the sum of each question and ranges from zero to one hundred.

### Statistical Analysis

Non-parametric analyses were used, as all data sets did not pass the Shapiro–Wilk test for normality (*p* < 0.05); Mann–Whitney test for each variable was performed on raw data to exclude significant differences at baseline (sham vs. active tsDCS). tsDCS-induced changes in each variable were then assessed by using the Friedman test (non-parametric analysis on paired data) with the main factor “time” (four levels: T0, T1, T2, and T3). In order to disclose significant changes at each time point between anodal and sham tsDCS, a Wilcoxon matched-pairs signed test was then applied. Both the electrophysiological measures and clinical scores were normalized to T0 score before entering the analysis [according to the formula (T1 – T0)/T0 ^*^ 100 + 100]. Finally, the Spearman's rank correlation coefficient was used to compare changes in electrophysiological parameters (LLFR) with the clinical outcome. Statistical significance was set at *p* < 0.05. The data were analyzed using SPSS v. 21.0 for Windows (SPSS Inc.). Neurophysiological and clinical data are expressed as mean ± Standard Error (S.E.).

## Results

### Neurophysiological Assessment

**Figure 2:**
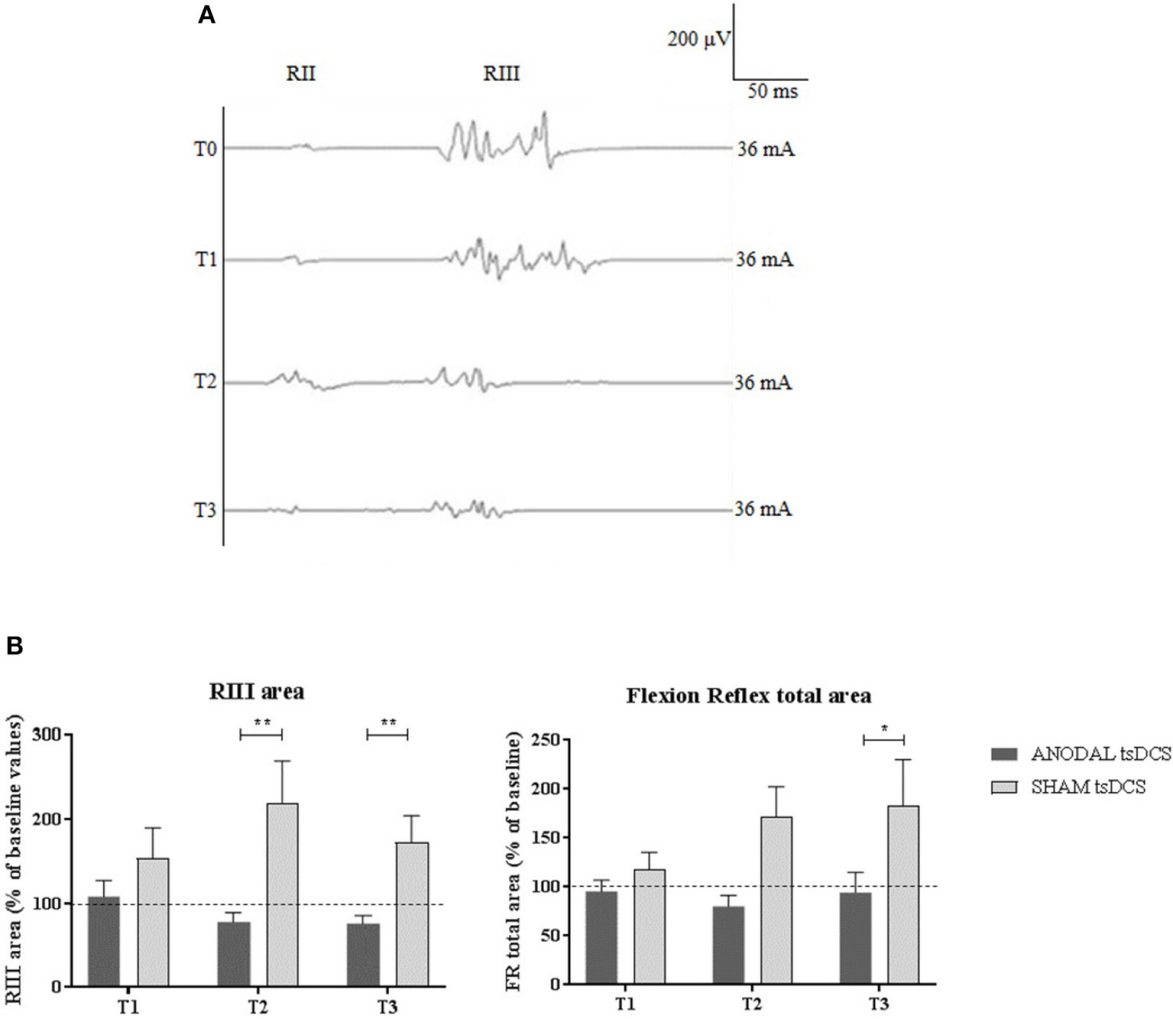
LLFR changes. **(A)** Exemplificative averaged traces of RIII reflex recorded before (T0), at the end of 5 days treatment (T1), and 1 week (T2) and 1 month (T3) after the completion of anodal tsDCS protocol. **(B)** Changes in RIII area and FR total area between anodal (black bars) and sham (gray bars) conditions. Data are given as normalized to T0 mean ± SE. The statistical significance refers to the comparison between anodal (active) and sham (placebo) stimulation (*p < 0.05; **p < 0.001, Wilcoxon's test *post-hoc* analysis).

Mann–Whitney test did not disclose any significant difference between the two groups at baseline, for each variable assessed (*p* > 0.05). The statistical analysis disclosed that changes both in RIII and FR total area between the two groups were found over time, with anodal tsDCS leading to a marked reduction of both parameters and sham condition eliciting opposite effects (Friedman's test: *p* < 0.0001 for RIII and *p* = 0.027 for FR total area) (Table 2; Figure 2). Regarding RIII area, this reduction was observed at T2 (Wilcoxon's test, as *post-hoc* analysis: *p* = 0.0043) and T3 (*p* = 0.0012); FR total area significantly decreased in the anodal compared to sham tsDCS at T3 (Wilcoxon's test: *p* = 0.03). The sham group showed a significant increase in RII latency from T0 to T3 (Friedman test: *p* < 0.0001; T0 vs. T3: *p* < 0.0001) and in RIII area from T0 to T2 (Friedman test: *p* < 0.01; T0 vs. T2: *p* < 0.01; Table 2; Figure 2).

**Table 2:** Neurophysiological results.

	**Anodal tsDCS**				**Sham tsDCS**			
	**T0**	**T1**	**T2**	**T3**	**T0**	**T1**	**T2**	**T3**
RIII threshold	36.17 ± 4.35	34.68 ± 5.06	31.20 ± 4.12	30.50 ± 4.96	34.59 ± 5.45	34.50 ± 5.8	31.85 ± 5.43	35.45 ± 7.66
RII latency	77.28 ± 2.97	83.38 ± 3.48	83.88 ± 3.81	80.05 ± 4.04	65.72 ± 6.43	69.37 ± 6.27	70.72 ± 6.39	70.04 ± 7.34
RIII latency	142.52 ± 6.10	142.38 ± 7.34	135.32 ± 5.47	135.08 ± 5.05	146.48 ± 6.18	137.87 ± 4.35	144.11 ± 5.54	147.69 ± 5.29
RIII area	5.96 ± 1.40	5.40 ± 1.25	4.18 ± 1.41	4.51 ± 1.04	4.71 ± 0.94	6.35 ± 1.95	7.29 ± 1.61	6.39 ± 1.41
FR total area	8.33 ± 1.64	7.18 ± 1.35	6.17 ± 1.83	6.68 ± 1.32	6.42 ± 1.20	6.83 ± 2.31	7.50 ± 1.85	8.19 ± 1.61

*RIII Threshold, RII Latency, RIII latency, RIII area, and FR total area are reported at T0 and over time, according to the group (anodal or sham tsDCS). Values are expressed in μV, as normalized to T0 mean values ± standard error (S.E)*.

### Clinical Outcome

**Figure 3:**
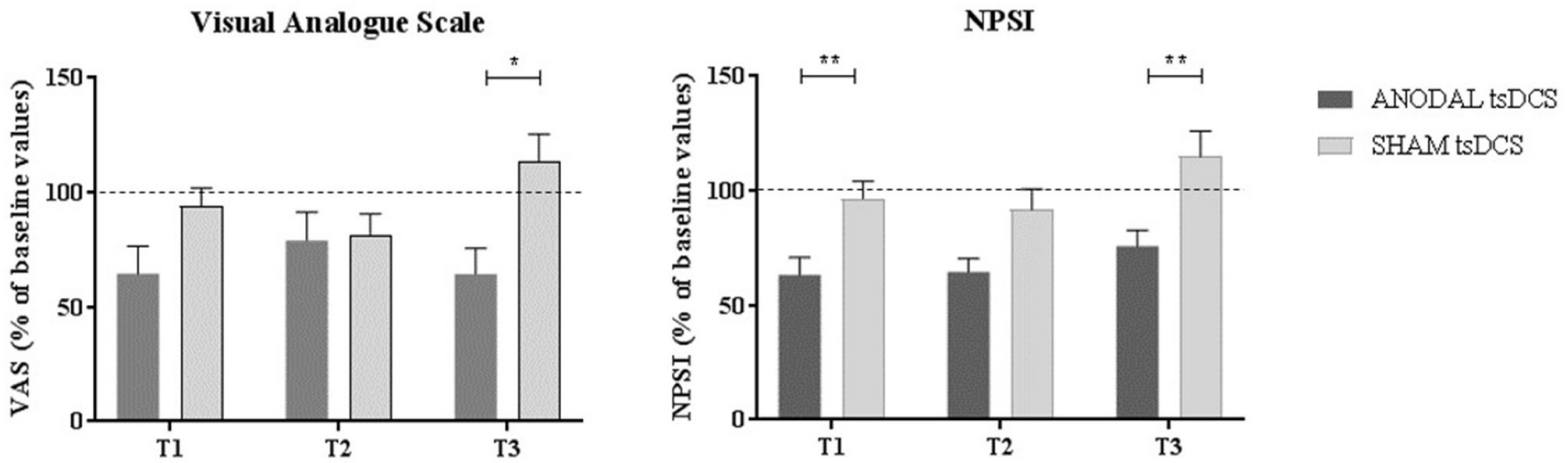
Clinical assessment of pain changes. Left: VAS score changes over time. Right: NPSI score changes over time. Data are given as normalized to T0 mean ± SE. The statistical significance refers to the comparison between anodal (active) and sham (placebo) stimulation (*p < 0.05; **p < 0.001, Wilcoxon's test *post-hoc* analysis).

Mann–Whitney test for each raw clinical variable (sham vs. anodal tsDCS) at baseline revealed no statistical difference between the two groups (*p* > 0.05). The active group significantly changed over time in VAS (Friedman test: *p* < 0.05) and NPSI (Friedman test: *p* < 0.0001) scores, with *post-hoc* pairwise comparison showing a significant reduction for NPSI from T0 to T1 (*p* < 0.0001) and from T0 to T2 (*p* < 0.0001). In sham condition, VAS showed significant changes over time, with scores increasing from T2 to T3 (Friedman test: *p* < 0.01; T2 vs. T3: *p* < 0.01), paralleled by a worsening of NPSI scores (Friedman test: *p* < 0.01; T2 vs. T3: *p* < 0.01) (Table 3; Figure 3). When compared the two stimulation conditions at each time point, VAS was significantly dampened at T3 (*p* = 0.015, Wilcoxon's), whereas NPSI scores improved both at T1 (*p* = 0.0012) and T3 (*p* = 0.0015). Changes in VAS and NPSI scores linearly correlated with the reduction in LLFR areas. Indeed, patients with greater clinical improvement showed a more robust modulation of neurophysiological responses (*p* = 0.0004, Spearman's rank correlation coefficient).

**Table 3:** Clinical assessment.

	**Anodal tsDCS**				**Sham tsDCS**			
	**T0**	**T1**	**T2**	**T3**	**T0**	**T1**	**T2**	**T3**
VAS	66.33 ± 6.85	41.40 ± 9.07	48.13 ± 7.6	41.75 ± 9.72	60.64 ± 7.67	57.82 ± 9.75	50.10 ± 10.62	63.80 ± 7.91
NPSI	43.20 ± 5.28	27.07 ± 5	29.2 ± 5.58	34.25 ± 7.51	36.36 ± 5.63	36.27 ± 7.09	32.20 ± 6.38	41.43 ± 7.94

*VAS and NPSI scores for anodal or sham tsDCS over time. Values are expressed in mm, as mean values ± standard error (S.E)*.

## Discussion

Our results demonstrated that the use of tsDCS for chronic pain subjects is safe and feasible. All subjects tolerated the stimulation well without dropouts. Anodal tsDCS dampened both RIII and FR total area and improved NPSI scores over time, thus resulting in an overall analgesic effect.

Anodal tsDCS modulates the nociceptive component (RIII) of the LLFR. These findings are in line with the results reported by Cogiamanian et al. (19), showing a significant reduction of RIII area by ~27% after anodal thoracic tsDCS in healthy subjects. In our study, we found a range of RIII area reduction higher than in healthy subjects. The RIII reflex is considered as the most reliable nociceptive reflex to assess the efficacy of pain treatments (27, 44), and it has been shown to be consistently hyperexcitable in some chronic pain conditions, including fibromyalgia (45, 46), Wallenberg's Syndrome (47), and some types of chronic headaches (48–50).

Moreover, it is important to note the high variability in our estimates (expressed by large standard errors). This is partially explained by our small sample size, and also due to the mixed etiologies of chronic pain we included. The overall neurophysiological results reported on neuropathic and chronic pain tend to be heterogeneous, which might suggest RIII changes to be pathophysiology dependent (27). Another important source of heterogeneity might come from the different pharmacological therapies. Results from tDCS studies clearly suggest that several pharmacological classes interfere with neuromodulation with a complex non-linear interaction (51, 52). Therefore, medications may tarnish tDCS effects themselves (51). For example, GABA modulators (e.g., Lorazepam) induced a delayed but prolonged increase of the excitability after anodal tDCS (53), while glutamatergic antagonist agents (e.g., dextromethorphan) abolish the after-effects of both anodal and cathodal tDCS (54, 55).

It has been suggested that RIII reflex variables are correlated with pain-related measurements. For instance, the threshold of the RIII reflex has been shown to correspond to the pain threshold and the area of the reflex to be related to the level of pain perception (27, 56). Our results support this hypothesis, since we found a pain intensity reduction in patients with small RIII area in the anodal condition. The within-group pain reduction was higher and consistent in the anodal tsDCS condition with a range of reduction from −18.2 to −24.58 mm in VAS, which are considered around the minimum change reflecting clinical differences in pain outcome (57). However, the difference between stimulations was not always significant after multiple comparison adjustment. This could be explained by different factors, such as the small sample size, the short duration of the stimulation protocol (5 days), or the heterogeneity of pain etiologies. Indeed, the need of a protocol optimization to identify the most effective tsDCS stimulation scheme for chronic pain (58) has been reported, considering also the different degrees of central sensitization (59) involved in such condition.

Finally, the lack of changes in RII parameters is not surprising, as this component reflects the activation of non-nociceptive, large-diameter fibers, although recent studies have suggested that Aβ terminals are also engaged in the pathophysiology of paroxysmal pain (60, 61).

The pain modulation mechanism of tsDCS remains open to questions. It has been suggested that anodal spinal tsDCS may produce a combination of spinal and supraspinal effects (62). Furthermore, anodal tsDCS can reduce the pain sensitivity associated with nociceptive mechanical stimuli (63) and can modulate temporal summation of pain modulating the activity of wide-dynamic-range neurons (64). Thus, tsDCS could modulate neuronal activity in lemniscal, spinothalamic, and segmental spinal circuits, suggesting glutamatergic, GABAergic, and glycinergic networks involvement (17). Then, tsDCS may ultimately interfere with supra-spinal mechanisms of pain sensitization, comprising the thalamo-cortical dysrhythmia and the maladaptive reorganization of cortical sensorimotor maps. Considering these potential mechanisms of action, we selected the position of the reference electrode in the somatosensory cortex. Parazzini et al. (65) estimated, based on current density simulation, that a cortical reference (attached to Cz) produces a higher magnitude of the current density in the cervical and thoracic regions, and it could improve its physiological effects by the site of action at both the spinal and supraspinal levels. Assuming the expected pain-related maladaptive plasticity in spinal and supraspinal levels, a montage with a cortical reference electrode was applied to maximize the neuroplastic effects of tsDCS in this study.

Moreover, Truini et al. (15) showed a significant decrease of N1 and N2 amplitude of laser evoked potentials (LEP) over the foot, but not over the perioral region after anodal tsDCS over T10. In line with these results, Lenoir et al. (66) reported significant effects of anodal T10 tsDCS over SEP-N2 wave elicited by nociceptive LEP over the foot, but not over the hand nor by non-nociceptive stimuli, suggesting that tsDCS modulatory effects are basically local and influence only the conduction of pain-stimulus coming below the stimulation site. Based on these, it is reasonable to consider the potential influences of the spinal cord injury locations in the design of future studies, as cervical or upper thoracic lesions might not respond the same way as lower thoracic lesions or lesions near the stimulation site.

This study has some limitations. First, the small sample size might have underpowered the study, even though we found neurophysiological and clinically significant results. Second, we included a heterogenous chronic pain population, with multiple neuropathic pain etiologies. The different degree of central nociceptive signal transmission affectation underlying such conditions might have affected the results and reduced the external validity of our findings. Similarly, our patients had stable but different (or even no) pharmacological therapies in place during the study. The different biochemical effects induced by the medications may have affected tsDCS effects, as previously suggested (51, 52).

In conclusion, our findings on anodal tsDCS-induced reduction of nociceptive reflex (RIII reflex area) and pain intensity (although marginally significant) in chronic pain subjects confirm that a non-invasive spinal neuromodulatory technique could modulate central nociceptive signal transmission and pain perception. Thus, tsDCS represents a promising intervention to reduce chronic pain by targeting pain-maladaptive plasticity. Further studies are needed with larger sample sizes, including a more homogenous population, and with optimized study design (double-blinded, parallel design) and stimulation protocols (longer stimulation protocols, concomitant interventions, spinal, and supraspinal biomarkers).

## Data Availability Statement

The raw data supporting the conclusions of this article will be made available by the authors, without undue reservation.

## Ethics Statement

The studies involving human participants were reviewed and approved by Ethics Committee of the IRCCS Ca' Granda Foundation - Maggiore Policlinico Hospital of Milan. The patients/participants provided their written informed consent to participate in this study.

## Author Contributions

MV and SB: concept and design. MV, GA, AN, and SV: acquisition of data. MV and TB: interpretation of data. MG, RF, KP-B, SG-L, and TB: drafting of the manuscript. AP: critical revision of the manuscript. All authors contributed to the article and approved the submitted version.

## Conflict of Interest

RF, MV, SB, and AP are founders and shareholders of Newronika Spa. The remaining authors declare that the research was conducted in the absence of any commercial or financial relationships that could be construed as a potential conflict of interest.

## Publisher's Note

All claims expressed in this article are solely those of the authors and do not necessarily represent those of their affiliated organizations, or those of the publisher, the editors and the reviewers. Any product that may be evaluated in this article, or claim that may be made by its manufacturer, is not guaranteed or endorsed by the publisher.

## References

[1] Mills SEE Nicolson KP Smith BH. Chronic pain: a review of its epidemiology and associated factors in population-based studies. Br J Anaesth. (2019) 123:e273–83. 10.1016/j.bja.2019.03.023 31079836 PMC6676152

[2] Yarnitsky D. Role of endogenous pain modulation in chronic pain mechanisms and treatment. Pain. (2015) 156:S24–31. 10.1097/01.j.pain.0000460343.46847.58 25789433

[3] Suzuki R Dickenson A. Spinal and supraspinal contributions to central sensitization in peripheral neuropathy. Neurosignals. (2005) 14:175–81. 10.1159/000087656 16215300

[4] Drolet M Brisson M Schmader K Levin M Johnson R Oxman M. Predictors of postherpetic neuralgia among patients with herpes zoster: a prospective study. J Pain. (2010) 11:1211–21. 10.1016/j.jpain.2010.02.020 20434957

[5] Flor H. Phantom-limb pain: characteristics, causes, and treatment. Lancet Neurol. (2002) 1:182–9. 10.1016/s1474-4422(02)00074-1 12849487

[6] Alshelh Z di Pietro F Youssef AM Reeves JM Macey PM Russell Vickers E. Chronic neuropathic pain: it's about the rhythm. J Neurosci. (2016) 36:1008–18. 10.1523/JNEUROSCI.2768-15.2016 26791228 PMC6602000

[7] Bocci T De Carolis G Ferrucci R Paroli M Mansani F Priori A. Cerebellar transcranial direct current stimulation (ctDCS) ameliorates phantom limb pain and non-painful phantom limb sensations. Cerebellum. (2019) 18:527–35. 10.1007/s12311-019-01020-w 30830672

[8] Bocci T Santarcangelo E Vannini B Torzini A Carli G Ferrucci R. Cerebellar direct current stimulation modulates pain perception in humans. Restor Neurol Neurosci. (2015) 33:597–609. 10.3233/RNN-140453 25777683

[9] Fregni F El-Hagrassy MM Pacheco-Barrios K Carvalho S Leite J Simis M. Evidence-based guidelines and secondary meta-analysis for the use of transcranial direct current stimulation (tDCS) in neurological and psychiatric disorders. Int J Neuropsychopharmacol. (2021) 24:256–313. 10.1093/ijnp/pyaa051 32710772 PMC8059493

[10] Fregni F Gimenes R Valle AC Ferreira MJL Rocha RR Natalle L. A randomized, sham-controlled, proof of principle study of transcranial direct current stimulation for the treatment of pain in fibromyalgia. Arthritis Rheum. (2006) 54:3988–98. 10.1002/art.22195 17133529

[11] Antal A Terney D Kühnl S Paulus W. Anodal transcranial direct current stimulation of the motor cortex ameliorates chronic pain and reduces short intracortical inhibition. J Pain Symptom Manage. (2010) 39:890–903. 10.1016/j.jpainsymman.2009.09.023 20471549

[12] Brunoni AR Amadera J Berbel B Volz MS Rizzerio BG Fregni F. A systematic review on reporting and assessment of adverse effects associated with transcranial direct current stimulation. Int J Neuropsychopharmacol. (2011) 14:1133–45. 10.1017/S1461145710001690 21320389

[13] Lefaucheur JP André-Obadia N Antal A Ayache SS Baeken C Benninger DH. Evidence-based guidelines on the therapeutic use of repetitive transcranial magnetic stimulation (rTMS). Clin Neurophysiol. (2014) 125:2150–206. 10.1016/j.clinph.2014.05.021 25034472

[14] Cogiamanian F Vergari M Pulecchi F Marceglia S Priori A. Effect of spinal transcutaneous direct current stimulation on somatosensory evoked potentials in humans. Clin Neurophysiol. (2008) 119:2636–40. 10.1016/j.clinph.2008.07.249 18786856

[15] Truini A Vergari M Biasiotta A La Cesa S Gabriele M Di Stefano G. Transcutaneous spinal direct current stimulation inhibits nociceptive spinal pathway conduction and increases pain tolerance in humans. Eur J Pain. (2011) 15:1023–7. 10.1016/j.ejpain.2011.04.009 21576030

[16] Ahmed Z. Trans-spinal direct current stimulation modulates motor cortex-induced muscle contraction in mice. J Appl Physiol. (2011) 110:1414–24. 10.1152/japplphysiol.01390.2010 21350028

[17] Ahmed Z Wieraszko A. Trans-spinal direct current enhances corticospinal output and stimulation-evoked release of glutamate analog, D-2,3- ^3^ H-aspartic acid. J Appl Physiol. (2012) 112:1576–92. 10.1152/japplphysiol.00967.2011 22362399

[18] Aguilar J Pulecchi F Dilena R Oliviero A Priori A Foffani G. Spinal direct current stimulation modulates the activity of gracile nucleus and primary somatosensory cortex in anaesthetized rats. J Physiol. (2011) 589:4981–96. 10.1113/jphysiol.2011.214189 21825031 PMC3224887

[19] Cogiamanian F Vergari M Schiaffi E Marceglia S Ardolino G Barbieri S. Transcutaneous spinal cord direct current stimulation inhibits the lower limb nociceptive flexion reflex in human beings. Pain. (2011) 152:370–5. 10.1016/j.pain.2010.10.041 21159430

[20] Lamy J-CC Ho C Badel A Arrigo RT Boakye M. Modulation of soleus H reflex by spinal DC stimulation in humans. J Neurophysiol. (2012) 108:906–14. 10.1152/jn.10898.2011 22623482

[21] Winkler T Hering P Straube A. Spinal DC stimulation in humans modulates post-activation depression of the H-reflex depending on current polarity. Clin Neurophysiol. (2010) 121:957–61. 10.1016/j.clinph.2010.01.014 20153248

[22] Bocci T Vannini B Torzini A Mazzatenta A Vergari M Cogiamanian F. Cathodal transcutaneous spinal direct current stimulation (tsDCS) improves motor unit recruitment in healthy subjects. Neurosci Lett. (2014) 578:75–9. 10.1016/j.neulet.2014.06.037 24970753

[23] Bocci T Marceglia S Vergari M Cognetto V Cogiamanian F Sartucci F. Transcutaneous spinal direct current stimulation modulates human corticospinal system excitability. J Neurophysiol. (2015) 114:440–6. 10.1152/jn.00490.2014 25925328 PMC4509392

[24] Bocci T Barloscio D Vergari M Di Rollo A Rossi S Priori A. Spinal direct current stimulation modulates short intracortical inhibition. Neuromodul Technol Neural Interface. (2015) 18:686–93. 10.1111/ner.12298 25880098

[25] Bocci T Caleo M Vannini B Vergari M Cogiamanian F Rossi S. An unexpected target of spinal direct current stimulation: interhemispheric connectivity in humans. J Neurosci Methods. (2015) 254:18–26. 10.1016/j.jneumeth.2015.07.012 26213216

[26] Willer JC Bathien N. Pharmacological modulations on the nociceptive flexion reflex in man. Pain. (1977) 3:111–9. 10.1016/0304-3959(77)90074-4 876670

[27] Sandrini G Serrao M Rossi P Romaniello A Cruccu G Willer JC. The lower limb flexion reflex in humans. Prog Neurobiol. (2005) 77:353–95. 10.1016/j.pneurobio.2005.11.003 16386347

[28] Ertekin C Ertekin N Karcioglu M. Conduction velocity along human nociceptive reflex afferent nerve fibres. J Neurol Neurosurg Psychiatry. (1975) 38:959–65. 10.1136/jnnp.38.10.959 1202167 PMC492130

[29] Cruccu G Sommer C Anand P Attal N Baron R Garcia-Larrea L. EFNS guidelines on neuropathic pain assessment: Revised (2009). Eur J Neurol. (2010) 17:1010–8. 10.1111/j.1468-1331.2010.02969.x 20298428

[30] Lundberg A. Multisensory control of spinal reflex pathways. Prog Brain Res. (1979) 50:11–28. 10.1016/S0079-6123(08)60803-1 121776

[31] Schomburg ED Petersen N Barajon I Hultborn H. Flexor reflex afferents reset the step cycle during fictive locomotion in the cat. Exp Brain Res. (1998) 122:339–50. 10.1007/s002210050522 9808307

[32] Jankowska E Jukes MGM Lund S Lundberg A. The effect of DOPA on the Spinal Cord 5. Reciprocal organization of pathways transmitting excitatory action to alpha motoneurones of flexors and extensors. Acta Physiol Scand. (1967) 70:369–88. 4293473 10.1111/j.1748-1716.1967.tb03636.x

[33] Lamy JC Boakye M. Seeking significance for transcutaneous spinal DC stimulation. Clin Neurophysiol. (2013) 124:1049–50. 10.1016/j.clinph.2013.01.007 23403262

[34] Lim CY Shin HI. Noninvasive DC stimulation on neck changes MEP. Neuroreport. (2011) 22:819–23. 10.1097/WNR.0b013e32834b939d 21915075

[35] Iyer MB Mattu U Grafman J Lomarev M Sato S Wassermann EM. Safety and cognitive effect of frontal DC brain polarization in healthy individuals. Neurology. (2005) 64:872–5. 10.1212/01.WNL.0000152986.07469.E9 15753425

[36] Nitsche MA Liebetanz D Lang N Antal A Tergau F Paulus W. Safety criteria for transcranial direct current stimulation (tDCS) in humans [1] (multiple letters). Clin Neurophysiol. (2003) 114:2220–2. 10.1016/s1388-2457(03)00235-9 14580622

[37] Galea JM Jayaram G Ajagbe L Celnik P. Modulation of cerebellar excitability by polarity-specific noninvasive direct current stimulation. J Neurosci. (2009) 29:9115–22. 10.1523/JNEUROSCI.2184-09.2009 19605648 PMC2760225

[38] Gandiga PC Hummel FC Cohen LG. Transcranial DC stimulation (tDCS): a tool for double-blind sham-controlled clinical studies in brain stimulation. Clin Neurophysiol. (2006) 117:845–50. 10.1016/j.clinph.2005.12.003 16427357

[39] Willer JC. Comparative study of perceived pain and nociceptive flexion reflex in man. Pain. (1977) 3:69–80. 876668 10.1016/0304-3959(77)90036-7

[40] Willer JC Le Bars D De Broucker T. Diffuse noxious inhibitory controls in man: Involvement of an opioidergic link. Eur J Pharmacol. (1990) 182:347–55. 10.1016/0014-2999(90)90293-F 2168836

[41] Sandrini G Arrigo A Bono G Nappi G. The nociceptive flexion reflex as a tool for exploring pain control systems in headache and other pain syndromes. Cephalalgia. (1993) 13:21–7. 8448783 10.1046/j.1468-2982.1993.1301021.x

[42] Collins SL Moore RA McQuay HJ. The visual analogue pain intensity scale: what is moderate pain in millimetres? Pain. (1997) 72:95–7. 10.1016/S0304-3959(97)00005-5 9272792

[43] Bouhassira D Attal N Fermanian J Alchaar H Gautron M Masquelier E. Development and validation of the neuropathic pain symptom inventory. Pain. (2004) 108:248–57. 10.1016/j.pain.2003.12.024 15030944

[44] Cruccu G Anand P Attal N Garcia-Larrea L Haanpaa M Jorum E. EFNS guidelines on neuropathic pain assessment. Eur J Neurol. (2004) 11:153–62. 10.1111/j.1468-1331.2004.00791.x 15009162

[45] Desmeules JA Cedraschi C Rapiti E Baumgartner E Finckh A Cohen P. Neurophysiologic evidence for a central sensitization in patients with fibromyalgia. Arthritis Rheum. (2003) 48:1420–9. 10.1002/art.10893 12746916

[46] Banic B Petersen-Felix S Andersen OK Radanov BP Villiger PM Arendt-Nielsen L. Evidence for spinal cord hypersensitivity in chronic pain after whiplash injury and in fibromyalgia. Pain. (2004) 107:7–15. 10.1016/j.pain.2003.05.001 14715383

[47] De Broucker TH Cesaro P Willer JC Le Bars D. Diffuse noxious inhibitory controls in man: involvement of the spinoreticular tract. Brain. (1990) 113:1223–34. 10.1093/brain/113.4.1223 2397390

[48] Langemark M Bach FW Jensen TS Olesen J. Decreased nociceptive flexion reflex threshold in chronic tension-type headache. Arch Neurol. (1993) 50:1061–4. 10.1001/archneur.1993.00540100056015 8215965

[49] Sandrini G Antonaci F Lanfranchi S Milanov I Danilov A Nappi G. Asymmetrical reduction of the nociceptive flexion reflex threshold in cluster headache. Cephalalgia. (2000) 20:647–52. 10.1111/j.1468-2982.2000.00096.x 11128822

[50] Antonaci F Sandrini G Danilov A Sand T. Neurophysiological studies in chronic paroxysmal hemicrania and hemicrania continua. Headache J Head Face Pain. (1994) 34:479–83. 10.1111/j.1526-4610.1994.hed3408479.x 7960734

[51] Brunoni AR Nitsche MA Bolognini N Bikson M Wagner T Merabet L. Clinical research with transcranial direct current stimulation (tDCS): challenges and future directions. Brain Stimul. (2012) 5:175–95. 10.1016/j.brs.2011.03.002 22037126 PMC3270156

[52] McLaren ME Nissim NR Woods AJ. The effects of medication use in transcranial direct current stimulation: a brief review. Brain Stimul. (2018) 11:52–8. 10.1016/j.brs.2017.10.006 29066167 PMC5729094

[53] Nitsche MA Liebetanz D Schlitterlau A Henschke U Fricke K Frommann K. GABAergic modulation of DC stimulation-induced motor cortex excitability shifts in humans. Eur J Neurosci. (2004) 19:2720–6. 10.1111/j.0953-816X.2004.03398.x 15147306

[54] Liebetanz D Nitsche MA Tergau F Paulus W. Pharmacological approach to the mechanisms of transcranial DC-stimulation-induced after-effects of human motor cortex excitability. Brain. (2002) 125:2238–47. 10.1093/brain/awf238 12244081

[55] Nitsche MA Fricke K Henschke U Schlitterlau A Liebetanz D Lang N. Pharmacological modulation of cortical excitability shifts induced by transcranial direct current stimulation in humans. J Physiol. (2003) 553:293–301. 10.1113/jphysiol.2003.049916 12949224 PMC2343495

[56] Rhudy JL France CR. Defining the nociceptive flexion reflex (NFR) threshold in human participants: a comparison of different scoring criteria. Pain. (2007) 128:244–53. 10.1016/j.pain.2006.09.024 17070999 PMC1993909

[57] Frahm Olsen M Bjerre E Hansen MD Tendal B Hilden J Hróbjartsson A. Minimum clinically important differences in chronic pain vary considerably by baseline pain and methodological factors: systematic review of empirical studies. J Clin Epidemiol. (2018) 101:87–106.e2. 10.1016/j.jclinepi.2018.05.007 29793007

[58] Pacheco-Barrios K Cardenas-Rojas A Thibaut A Costa B Ferreira I Caumo W. Methods and strategies of tDCS for the treatment of pain: current status and future directions. Expert Rev Med Dev. (2020) 17:879–98. 10.1080/17434440.2020.1816168 32845195 PMC7674241

[59] Bushnell MC Ceko M Low LA. Cognitive and emotional control of pain and its disruption in chronic pain. Nat Rev Neurosci. (2013) 14:502–11. 10.1038/nrn3516 23719569 PMC4465351

[60] Truini A Padua L Biasiotta A Caliandro P Pazzaglia C Galeotti F. Differential involvement of A-delta and A-beta fibres in neuropathic pain related to carpal tunnel syndrome. Pain. (2009) 145:105–9. 10.1016/j.pain.2009.05.023 19535205

[61] Farina S Valeriani M Rosso T Aglioti S Tamburin S Fiaschi A. Transient inhibition of the human motor cortex by capsaicin-induced pain. A study with transcranial magnetic stimulation. Neurosci Lett. (2001) 314:97–101. 10.1016/s0304-3940(01)02297-2 11698155

[62] Cogiamanian F Ardolino G Vergari M Ferrucci R Ciocca M Scelzo E. Transcutaneous spinal direct current stimulation. Front Psychiatry. (2012) 3:63. 10.3389/fpsyt.2012.00063 22783208 PMC3389353

[63] Meyer-Frießem CH Haag LM Schmidt-Wilcke T Magerl W Pogatzki-Zahn EM Tegenthoff M. Transcutaneous spinal DC stimulation reduces pain sensitivity in humans. Neurosci Lett. (2015) 589:153–8. 10.1016/j.neulet.2015.01.029 25596439

[64] Perrotta A Bolla M Anastasio MG Serrao M Sandrini G Pierelli F. Modulation of temporal summation threshold of the nociceptive withdrawal reflex by transcutaneous spinal direct current stimulation in humans. Clin Neurophysiol. (2016) 127:755–61. 10.1016/j.clinph.2015.01.031 25777061

[65] Parazzini M Fiocchi S Liorni I Rossi E Cogiamanian F Vergari M. Modeling the current density generated by transcutaneous spinal direct current stimulation (tsDCS). Clin Neurophysiol. (2014) 125:2260–70. 10.1016/j.clinph.2014.02.027 24784477

[66] Lenoir C Jankovski A Mouraux A. Anodal transcutaneous spinal direct current stimulation (tsDCS) selectively inhibits the synaptic efficacy of nociceptive transmission at spinal cord level. Neuroscience. (2018) 393:150–63. 10.1016/j.neuroscience.2018.10.007 30321585 PMC6364802

